# A gallbladder torsion presenting as acute cholecystitis in an elderly woman: A case report

**DOI:** 10.1186/1752-1947-5-588

**Published:** 2011-12-20

**Authors:** Jorine Boer, Djamila Boerma, Tammo S de Vries Reilingh

**Affiliations:** 1 Department of Surgery, St. Antonius Hospital, Postbus 2500, 3430 EM Nieuwegein, The Netherlands; 2 Department of Surgery, Elkerliek Hospital, Postbus 98, 5700 AB Helmond, The Netherlands

## Abstract

**Introduction:**

Gallbladder torsion is a rare, but potentially lethal disease, in which early recognition is crucial.

**Case presentation:**

We describe the case of an 89-year-old Caucasian woman who presented with clinical symptoms suggestive of acute cholecystitis to our hospital. Radiological imaging confirmed our clinical diagnosis. At first we considered percutaneous gallbladder drainage because of her age and comorbidity, but instead performed laparoscopic cholecystectomy because of rapid clinical deterioration. During laparoscopy a necrotic gallbladder due to torsion of the gallbladder around the cystic duct was found.

**Conclusion:**

Because percutaneous drainage could lead to further deterioration in the case of gallbladder torsion, this rare condition should be considered before performing a percutaneous drainage of cholecystitis.

## Introduction

Gallbladder torsion is a rare but potentially fatal condition only a few cases of which have been reported in the recent literature [1,2]. The majority of the described cases occurred in elderly women in the sixth and eighth decades of life [3]. Although etiology of gallbladder torsion is unknown, several predisposing factors have been recognized, including variations in hepatobiliary anatomy. Most cases are found coincidentally during surgery, as the clinical presentation is often suggestive of acute cholecystitis and the disease is fairly unknown [4]. We present a case of gallbladder torsion and review the clinical aspects of the disease.

## Case presentation

**Figure 1:**
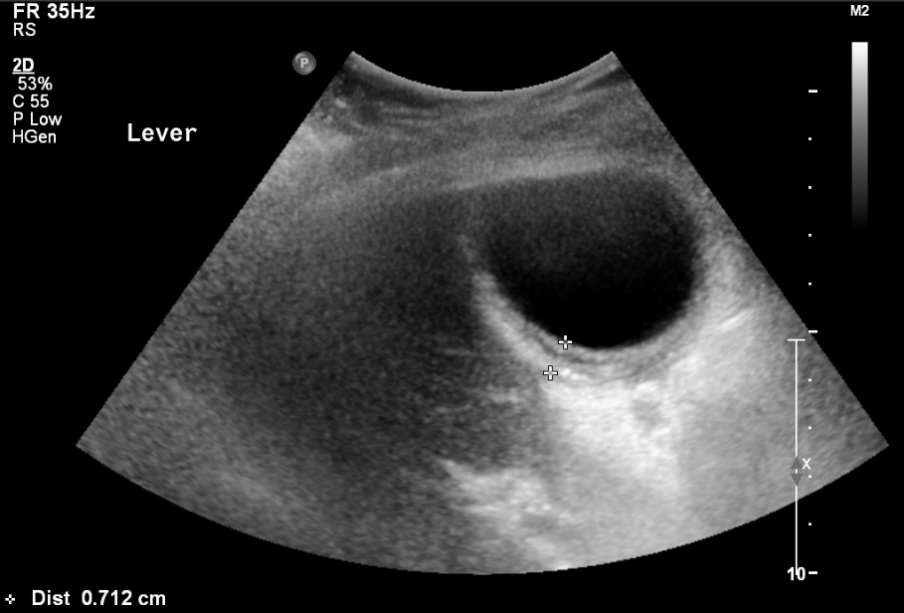
**Ultrasound image of the gallbladder**. Ultrasound image of the gallbladder acquired at presentation of the patient in the emergency department. The gallbladder is shown with a thickened wall (7 mm) suggesting inflammation.

**Figure 2:**
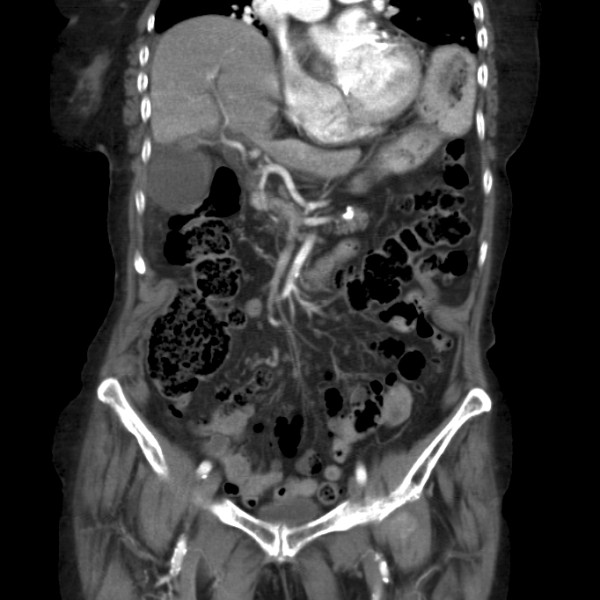
**CT scan of the abdomen**. Coronal view of the abdomen, acquired using a computed tomography scan with intravenous contrast, at presentation of the patient in the emergency department. The gallbladder is hydropic, with a thickened wall and positioned adjacent to the lateral abdominal wall. No gallstones are visible. Some isolated free fluid is seen between the right liver lobe and the diaphragm, as well as some fluid and fatty infiltration around the gallbladder. Radiological findings are suggestive of an acalculous cholecystitis.

An 89-year-old Caucasian woman presented to the emergency department with a one day history of acute onset abdominal pain in the upper right quadrant, with nausea and malaise. On presentation her vital signs were within the normal range and she had no fever. Her medical history included an abdominal rectopexy, retropubic bladder suspension surgery and an abdominal hysterectomy, all performed more than two decades ago. Physical examination showed an abdomen with a scar after median laparotomy with a palpable tender mass in the upper right quadrant with positive Murphy's sign and rebound tenderness. Laboratory blood tests revealed a leukocytosis of 22.7 × 10^9^/L, C-reactive protein of 48 mg/L and normal kidney and liver function tests. Abdominal ultrasonography and computed tomography (CT) scan showed a clearly enlarged gallbladder with a thickened wall of 7 mm (Figure 1), with fluid supra- and sub-hepatically (Figure 2). Free air within the gallbladder wall was not seen. She was admitted to our hospital with the diagnosis of acute cholecystitis.

**Figure 3:**
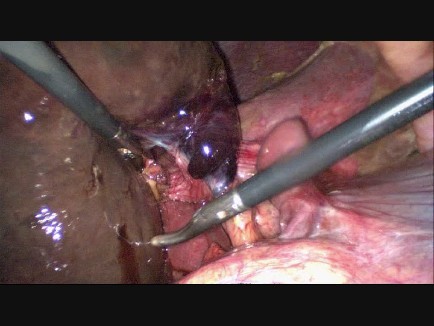
**Necrotic gallbladder**. Peroperative endoscopic image showing the gangrenous gallbladder with necrosis extending into the cystic duct and artery.

**Figure 4:**
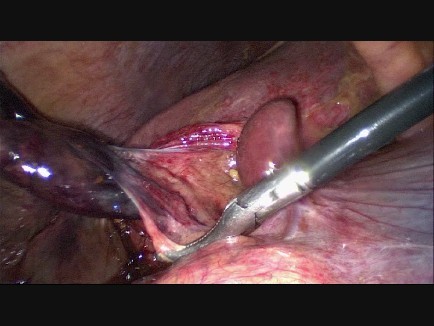
**Torsion of the gallbladder**. Peroperative endoscopic image showing a gangrenous gallbladder, rotated around the cystic duct and artery.

Percutaneous drainage of the gallbladder was considered because of her age and comorbidity, but because of her rapid clinical deterioration we decided to perform a laparoscopic cholecystectomy instead. Laparoscopy was performed and revealed a strongly hydropic and fully necrotic gallbladder, with necrosis extending into the cystic duct (Figure 3). The gallbladder was not embedded in the liver, but was hanging from the cystic duct and artery. Further exploration showed a two-fold torsion of the gallbladder around the cystic duct (Figure 4). A cholecystectomy was performed. (See Additional file 1 for further imaging of peroperative findings). As the critical level of safety was obtained, with both the cystic and hepatic duct clearly visible, leaving a drain was not considered mandatory. Pathologic examination revealed the gangrenous aspect of the entire gallbladder, extending into the cystic duct. No gallbladder stones were found in the specimen. Postoperatively the patient recovered well and she was discharged from the hospital on the fifth postoperative day.

## Discussion

Percutaneous drainage of acute cholecystitis is increasingly considered an alternative to laparoscopy in the elderly with high technical and clinical success rates of up to 98% [5,6]. Consequently, percutaneous drainage is being increasingly used as a treatment in high-risk patients with acute cholecystitis. If our patient had been treated by percutaneous drainage, this would have only treated the symptoms and, as the torsion would continue to exist, progressive ischemia with perforation of the gallbladder, progressive sepsis and possibly death could have occurred [7,8].

It is possible that the presence of gallbladder torsion instead of acute cholecystitis may account for some lack of clinical success of percutaneous cholecystostomy.

Gallbladder torsion, a condition in which the gallbladder rotates on its mesentery along the axis of the cystic duct and cystic artery, was first described in a 23-year-old woman by Wendell in 1898 [9]. Its incidence has been estimated at one in 365,000 hospital admissions [8,10,11]. Incidence seems to be increasing, most likely due to the increasing age of the population [11]. Although gallbladder torsion has been reported in patients ranging in age from two to 100 years there are two age groups of peak incidence: first between six to 19 years and second between 60 to 80 years with a male to female ratio of 4:1 in children and 1:3 in adults [2-4,8,10].

Although its precise etiology remains unknown, gallbladder torsion is believed to occur in anatomically predisposed patients. It is estimated that 4% to 5% of the population has a congenital anatomic anomaly predisposing to gallbladder torsion [1,3,8,11]. Four anatomic variants have been identified. The first anomaly consists of a free-floating gallbladder suspended only by the cystic duct and artery with an absent gallbladder mesentery, due to abnormal migration of the pars cystica from the hepatic diverticulum during the fourth to seventh weeks of embryological development [11,12]. Secondly, atrophy of the liver in combination with decreased elasticity of connective tissue in an elderly patient with a normally formed mesentery can lead to a progressively mobile gallbladder [2,3,10,11]. Thirdly, rotation of a portion of the fundus may occur, when it is not fixed to the liver bed [10,13]. Finally, a normal fixed gallbladder can rarely be attached to a mobile hepatic lobe free of its coronary and triangular ligaments, therefore allowing torsion [2,11,13]. Pathophysiological factors which have been suggested to precipitate torsion in predisposed individuals are kyphoscoliosis, generalized visceroptosis, adhesions, sudden body movements, iatrogenic manipulation of the abdomen, blunt abdominal trauma, vigorous peristalsis of neighboring organs, malnutrition, heavy meals, weight loss, constipation, diarrhea, multiparity and postpartum state [1,4,10,11,13]. The presence of gallstones is not a risk factor as it is only reported in 20% to 33% of cases, where normal prevalence of asymptomatic gallstones can be as high as 22% [1].

Clinical presentation is diverse, ranging from acute abdomen to chest pain, but it most commonly presents as acute pain in the upper right quadrant. A retrospective study of 245 cases of gallbladder torsion reported abdominal pain as the most reported symptoms (100%), followed by vomiting (52.7%), a palpable mass in the upper right abdomen (32.6%) and fever (31.6%) [3]. Clinical presentation may point towards the diagnosis of acute cholecystitis, but some differences are useful to discriminate, such as absence of toxemia, absence of fever and rapid increase of symptoms.

Triple triads of clinical presentation have been proposed, consisting of symptoms (short history, abdominal pain and early vomiting), physical signs (abdominal mass, absence of toxemia and pulse-temperature discrepancy) and patients' characteristics (thin, elderly, deformed spine) [1,8,11]. Laboratory tests show unspecific inflammatory signs, with leukocytosis and raised C-reactive protein. Liver function tests are usually normal [3,10,12].

Preoperative diagnosis of gallbladder torsion is difficult, not in the least because it is a fairly unknown disease. Diagnosis was made before surgery in fewer than 10% of cases [3,14]. Radiological imaging can help with diagnosis. Ultrasound often shows a large gallbladder without gallstones, lying transversely with fluid in the gallbladder fossa, with a thickened wall and a conical appearance of the neck with discontinuity of lumen suggesting torsion [1-3,7,10,11,14]. It can also show a hypo-echoic zone between two echoic zones of the gallbladder, suggesting edema, most probably due to venous and lymphoid stasis. Duplex, although seldom used, can confirm the absence of flow through the cystic artery [3]. Computed tomography (CT) scan shows similar findings as ultrasound, with a fluid collection between the gallbladder and liver bed, a gallbladder positioned horizontally along its long axis, presence of a well-enhanced cystic duct located on the right side of the gallbladder and signs of inflammation, such as edema, indicating ischemia or necrosis [4,10,12,14,15]. A rare, but very specific sign on CT is the so-called 'whirl-sign'; only visible if the plane of CT is perpendicular to the axis of the twisted gallbladder mesentery [15]. MRI, sometimes used for diagnosis in children, can show high signal intensity within the gallbladder on T1-weighted images, suggestive of necrosis [8,10,14].

Although diagnostic techniques are becoming more sensitive, gallbladder torsion is still mostly diagnosed during surgery. Prompt cholecystectomy is recommended and a laparoscopic approach can be safely used in most cases. The objective of laparoscopy should be decompression and detorsion, followed by cholecystectomy [4,8,10]. When early diagnosis and treatment is achieved, mortality rates are around 3% to 5% [4,7,12].

Regarding the triple triads of clinical presentation as described above the diagnosis of gallbladder torsion should have been considered in our patient: she had seven out of nine signs, only lacking early vomiting and absence of toxemia. In retrospect the findings of radiological imaging also hinted at gallbladder torsion: showing a horizontal gallbladder with signs of inflammation and fluid between the gallbladder and liver bed, both radiological signs often found in gallbladder torsion. Radiological imaging did not show free air within the gallbladder wall, a common radiological finding when the gallbladder is necrotic. If this had been present, this would have supported the diagnosis of gangrenous cholecystitis, and thus torsion.

## Conclusions

Gallbladder torsion is a rare disease, which is still difficult to diagnose preoperatively despite advances in diagnostic imaging. It mainly affects elderly women and the clinical presentation is often suggestive of acute cholecystitis. Percutaneous drainage, as is often opted for in this patient group, can result in progressive necrosis followed by biliary peritonitis and even death. Therefore, it is important to be familiar with the clinical signs of gallbladder torsion and to consider the diagnosis before opting for percutaneous drainage for acute cholecystitis.

## Abbreviations

CT: computed tomography.

## Consent

Written informed consent was obtained from the patient for publication of this case report and any accompanying images. A copy of the written consent is available for review by the Editor-in-Chief of this journal.

## Competing interests

The authors declare that they have no competing interests.

## Authors' contributions

JB interpreted patient data, collected previous published literature on the subject and contributed in writing the manuscript. DB was also a contributor in writing the manuscript, as well as providing expert revision. TVR performed the surgical procedure, provided the endoscopic images and contributed in writing the manuscript. All authors read and approved the final manuscript.

## Supplementary Material

Additional file 1 **Torsion of the gallbladder**. Endoscopic recordings during inspection and laparoscopic removal of the gallbladder, showing a rotated gallbladder, with a gangrenous aspect of the gallbladder, the cystic duct and the cystic artery. The movie shows derotation of the gallbladder (0 to 23 s), followed by decompression (24 to 33 s), and cholecystectomy (34 to 42 s). Clipping of the hilum is not specifically shown, but two proximal and one distal clip are visible at 00:00:35. Click here for file
